# Novel Phylogenetic Algorithm to Monitor Human Tropism in Egyptian H5N1-HPAIV Reveals Evolution toward Efficient Human-to-Human Transmission

**DOI:** 10.1371/journal.pone.0061572

**Published:** 2013-04-26

**Authors:** Vladimir R. Perovic, Claude P. Muller, Henry L. Niman, Nevena Veljkovic, Ursula Dietrich, Dusan D. Tosic, Sanja Glisic, Veljko Veljkovic

**Affiliations:** 1 Center for Multidisciplinary Research, Institute of Nuclear Sciences Vinca, University of Belgrade, Belgrade, Serbia; 2 Institute of Immunology, Centre de Recherche Public de la Santé/Laboratoire National de Santé, Luxembourg, Grand-Duchy of Luxembourg; 3 Recombinomics, Inc., Pittsburgh, Pennsylvania, United States of America; 4 Georg-Speyer-Haus, Institute of Biomedical Research, Frankfurt-am-Main, Germany; 5 Faculty of Mathematics, University of Belgrade, Belgrade, Serbia; Institute of Molecular Genetics IMG-CNR, Italy

## Abstract

Years of endemic infections with highly pathogenic avian influenza (HPAI) A subtype H5N1 virus in poultry and high numbers of infections in humans provide ample opportunity in Egypt for H5N1-HPAIV to develop pandemic potential. In an effort to better understand the viral determinants that facilitate human infections of the Egyptian H5N1-HPAIVvirus, we developed a new phylogenetic algorithm based on a new distance measure derived from the informational spectrum method (ISM). This new approach, which describes functional aspects of the evolution of the hemagglutinin subunit 1 (HA1), revealed a growing group G2 of H5N1-HPAIV in Egypt after 2009 that acquired new informational spectrum (IS) properties suggestive of an increased human tropism and pandemic potential. While in 2006 all viruses in Egypt belonged to the G1 group, by 2011 these viruses were virtually replaced by G2 viruses. All of the G2 viruses displayed four characteristic mutations (D43N, S120(D,N), (S,L)129Δ and I151T), three of which were previously reported to increase binding to the human receptor. Already in 2006–2008 G2 viruses were significantly (p<0.02) more often found in humans than expected from their overall prevalence and this further increased in 2009–2011 (p<0.007). Our approach also identified viruses that acquired additional mutations that we predict to further enhance their human tropism. The extensive evolution of Egyptian H5N1-HPAIV towards a preferential human tropism underlines an urgent need to closely monitor these viruses with respect to molecular determinants of virulence.

## Introduction

H5N1-HPAIV emerged in Egypt in 2006 [1] and was declared endemic in 2008 [2]. Among the 181 human H5N1-HPAIV cases reported worldwide between 2009 and 2011, 58.6% occurred in this country alone [3]. Since Egypt became the new epicenter of human H5N1-HPAIV infections, the increasing pandemic potential of H5N1-HPAIV is a major concern. H5N1-HPAIV causes case fatality rates that are ten times higher than those seen during the 1918 flu pandemic but its infectivity is much lower. An infectivity of H5N1-HPAIV similar to seasonal flu would cause a disastrous pandemic. This threat is compounded by variants of H5N1-HPAIV that are less virulent and may even cause asymptomatic human infections [4]. Moreover an increasing infectivity of H5N1-HPAIV in humans paired with its silent spread in humans could further accelerate its evolution and adaptation to human-to-human transmission. Therefore there is an urgent need to better understand viral determinants that facilitate human infections by H5N1-HPAIV.

Entry of influenza virus into susceptible cells is mediated by the viral hemagglutinin (HA) membrane glycoprotein binding to its host cell receptor. Receptor binding selectivity of the HA protein correlates to some extent with the host range of the virus. We have recently used the informational spectrum method (ISM) to analyze the interaction of HA with its receptor [5], [6]. According to this virtual spectroscopy method, the amino acid sequence of a protein is transformed into a series of signals by assigning its electron-ion interaction potential (EIIP) value to each amino acid. The numerical series obtained is a (finite-length deterministic discrete) signal containing information corresponding to the selective long-range interaction between interacting proteins (interactions at distances >100 Å; for a review see Ref. [7] and [8]). The signal obtained is then decomposed into periodical functions by Fourier transformation. The result is a series of frequencies each with its own amplitude referred to as the Informational Spectrum (IS). This technique allows the detection or definition of amplitude/frequency pairs determining the specific long-range recognition between interacting proteins [7], [8]. The ISM approach has been applied to a large range of proteins including anthrax protective antigen, p53, HIV-1 envelope glycoprotein gp120, lipoprotein lipase, and others (for review see Ref. [7]). We have shown by ISM that influenza HA1 protein encodes, despite its variability, conserved information which characterizes recognition and targeting of its receptor. This information is represented by characteristic frequencies of IS of HA1 proteins. The comparison of the IS of similar sets of viruses that differ by a characteristic biological feature, e.g. their tropism or propensity to bind preferentially to a human instead of an animal host receptor permits to identify these characteristic frequencies. For instance, North American H1N1 viruses from pigs had a high amplitude at F(0.055) and a low amplitude at F(0.295), while the opposite was observed for pandemic H1N1 virus. Interestingly, pig viruses recovered from humans between 2005 and 2007 had a mixed profile. Thus a characteristic shift from the frequency F(0.055), characterizing the swine interacting profile, to the frequency F(0.295) corresponding to the human tropism, was documented in the evolution of swine H1N1 towards pandemic A(H1N1) [6].

In a similar study, we compared the IS of Egyptian H5N1-HPAIV with a broad selection of 28 seasonal H1N1 viruses isolated mainly from humans (n = 19) but also from swine (n = 5) and avian (n = 3) including also a mouse adapted laboratory strain. The differences in amplitudes showed that the frequency F(0.076) in the IS of HA1 corresponds to the preferred tropism of H5N1-HPAIV and the frequency F(0.236) characterizes the tropism of the seasonal H1N1 viruses [5]. The IS of most HA1 proteins from Egyptian H5N1-HPAIV contained both of these frequency components F(0.076) and F (0.236), but there was no IS similarity between H5N1 and seasonal H3N2 or pandemic H1N1 2009 influenza viruses. The preference for either one frequency can be described by the ratio between the amplitudes of the frequencies F(0.076) and F(0.236) (A(0.076) and A(0.236)) in the IS of H5N1-HPAIV HA1 [5]. We have speculated that a higher A(0.236)/A(0.076) ratio would correspond to a higher selectivity for the human receptor and a higher infectivity for humans [5].

Therefore, we extended our IS analysis of the evolution of H5N1 in Egypt to include all strains between 2006 and 2011. In order to further refine the IS analysis, we developed a phylogenetic approach based on critical IS features that determine the host tropism. We demonstrate that the IS-based phylogenetic analysis of HA1 revealed that H5N1-HPAIV circulating in Egypt after 2009 acquired specific structural and informational properties which may further increase their human-to-human transmission and pandemic potential.

## Materials and Methods

### Sequences

All of the 526 published sequences of H5N1-HPAIV isolated in Egypt between 2006 and 2011 were downloaded from NCBI [9] and GISAID [10] databases (Dataset S1) and submitted to phylogenetic analysis of HA1.

### Informational Spectrum Method (ISM)

The ISM is based on the assumption that the protein-protein interaction encompasses two basic steps: (i) recognition and targeting between interacting proteins (long-range interactions at distances >100 Å) and (ii) chemical binding (short range interactions at distances <5 Å) (reviewed in Ref. [7] and [8]). The long-range properties of biological molecules are determined by EIIP representing the main energy term of valence electrons [11]. The EIIP for organic molecules can be calculated by the following simple equation derived from the “general model pseudopotential” [12], [13]:



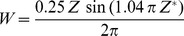
(1)where Z* is the average quasivalence number (AQVN) determined by
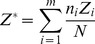
(2)where Z_i_ is the valence number of the i-th atomic component, n_i_ is the number of atoms of the i-th component, m is the number of atomic components in the molecule, and N is the total number of atoms. The EIIP values calculated according to equations (1) and (2) are expressed in Rydbergs (Ry) units.

The ISM assigns to each amino acid of the protein sequence its EIIP value (Table 1). This numerical sequence, corresponding to the protein sequence, is then subjected to a discrete Fourier transformation which is defined as follows:
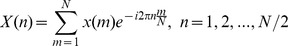
(3)where x(m) is the m-th member of a given numerical series, N is the total number of points in this series, and X(n) are discrete Fourier transformation coefficients. These coefficients describe the amplitude, phase and frequency of sinusoids which compose the original signal. The absolute values of a complex discrete Fourier transformation define the amplitude spectrum and the phase spectrum. The complete information about the original sequence is contained in both spectral functions. However, in the case of protein analysis, relevant information is presented in an energy density spectrum [14], which is defined as follows:
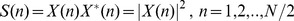
(4)


**Table 1:** The electron-ion interaction potential (EIIP) used to encode amino acids.

Amino acid	EIIP [Ry]
Leu	0.0000
Ile	0.0000
Asn	0.0036
Gly	0.0050
Glu	0.0057
Val	0.0058
Pro	0.0198
His	0.0242
Lys	0.0371
Ala	0.0373
Tyr	0.0516
Trp	0.0548
Gln	0.0761
Met	0.0823
Ser	0.0829
Cys	0.0829
Thr	0.0941
Phe	0.0946
Arg	0.0959
Asp	0.1263

In this way, sequences are analyzed as discrete signals. It is assumed that their points are equidistant with the distance d = 1. The maximal frequency in a spectrum defined as above is F = 1/2 d = 0.5. The frequency range is independent of the total number of points in the sequence. The total number of points in a sequence influences only the resolution of the spectrum. The resolution of the N-point sequence is 1/N. The n-th point in the spectral function corresponds to a frequency f(n) = nf = n/N. Thus, the initial information defined by the sequence of amino acids can now be presented in the form of the informational spectrum (IS), representing series of frequencies and their amplitudes.

The IS frequencies correspond to the distribution of structural motifs with defined physicochemical properties determining a biological function of a protein [7], [8]. When comparing proteins, which share the same biological or biochemical function, the ISM allows the detection of code/frequency pairs which are specific for their common biological properties, or which correlate with their specific interaction [7], [8]. These common informational characteristics of sequences are determined by cross-spectrum or consensus informational spectrum (CIS). A CIS of M spectra is obtained by the following equation:
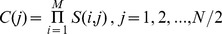
(5)where S(i,j) is the j-th element of the i-th power spectrum and C(j) is the j-th element of CIS. Thus, CIS is the Fourier transform of the correlation function for the spectrum. Thus, any spectral component (frequency) not present in all compared informational spectra is eliminated. Peak frequencies in CIS represent the common information encoded in the primary structure of the sequences analyzed. This information corresponds to the mutual long-range interaction between the proteins of interest or their interaction with the common interactor.

### Phylogenetic Analysis

Phylogeny determines the evolutionary relationship within a family of closely related sequences. Methods for constructing and analyzing phylogenetic trees can be divided into three groups [15], depending on the level of sequence similarity. The first group includes maximum parsimony methods [16], [17] used for quite similar sequences. The strategy is to minimize the number of evolutionary changes e.g. substitutions. The second group contains distance methods [18] used for sequences that still share recognizable sequence similarity. This approach is based on a distance matrix which represents the distance between each pair of sequences. The distance scores are determined from the alignment score [18] or uses various distance measure models [19], [20]. In all of these approaches evolutionary models are based on the multiple sequence alignment (MSA). The matrix is then transformed into a phylogenetic tree that represents genetic distances. The most common algorithms are the neighbor-joining method (NJ) [21] and the Unweighted Pair Group Method with Arithmetic Mean (UPGMA) [22]. The third group of methods uses a probabilistic approach to phylogeny. The trees are constructed by maximum likelihood methods [23] or sampling methods [24].

### A New Distance Measure for Phylogenetic Trees Based on IS

The main weakness of phylogenetic analyses based on MSA, is that sequence similarity does not necessarily imply similarity in functions. For example, two protein sequences that differ by a single mutation that is lethal for the biological function will nevertheless phylogenetically group together, whereas two proteins that differ in several mutations that do not affect biological functions will be phylogenetically separated. In order to overcome this obstacle and to improve functional sequence analysis, we propose a new distance measure based on the IS method.

For comparison of two sets of sequences of proteins with two different biological functions (here HA1 sequences from H5N1-HPAIV infecting birds and seasonal H1N1 viruses infecting humans) the first step is to identify the IS frequency components F_1_ and F_2_ that represent these two biological functions (here F_1_ = 0.076 corresponding to the tropism of the H5N1-HPAIV, and F_2_ = 0.236 corresponding to the tropism of the seasonal H1N1 virus). The characteristic frequencies F_1_ and F_2_ emerge as the dominant peaks A(F_1_) and A(F_2_) in the consensus IS calculated for each set of sequences as described above.

S_1_ and S_2_ are the IS spectra of the two sequences X_1_ and X_2_. A_1_(F_1_) and A_1_(F_2_) are the amplitudes of the two characteristic frequencies F_1_ and F_2_ of spectrum S_1_. Similarly, A_2_(F_1_) and A_2_(F_2_) are the amplitudes of the sequences F_1_ and F_2_ of spectrum S_2_. Then the distance between X_1_ and X_2_ is the absolute difference of the amplitude ratios:
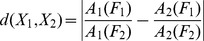
(6)


Let P be the set of values A(F_1_)/A(F_2_) for every sequence X, where A(F_1_) and A(F_2_) are spectrum amplitudes of sequence S, at the frequencies F_1_ and F_2_, respectively. Set P is a subset of the set of real numbers R. Distance d is then the Euclidean distance on R, and so it is a valid metric measure on P. It satisfies:

1. d(x,y)≥0, non-negativity
2. d(x,y) = 0 ⇔ x = y, identity of indiscernibles
3. d(x,y) = d(y,x), symmetry
4. d(x,z) ≤ d(x,y)+d(y,z), triangle inequality

It is also a valid additive evolutionary measure on P. It satisfies additivity (four point condition): two of three sums d(x,y)+d(z,w), d(x,z)+d(y,w), d(x,w)+d(y,z) are equal and larger than a third sum. The distance matrix based on the distance d was used to generate a phylogenetic tree using the following algorithm.

1. For each sequence calculate its spectrum
  (a) Convert amino acid sequence into signal with EIIP values
  (b) Decrease signal to zero mean
  (c) Zero-padding to length of the longest signal, to set the same resolution to all spectra
  (d) Apply Fast Fourier Transformation to signal to generate energy density spectrum
2. Calculate the cross-spectrum for each set of sequences to be compared
3. Determine two characteristic frequencies F_1_ and F_2_ e.g. those with the highest amplitude in these two cross-spectra
4. Calculate the distance matrix with the following distance measure
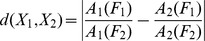

5. Construct the tree using the NJ method.

Of note is that the conventional likelihood or bootstrap validation are not applicable to our algorithm because the ISM phylogenetic approach is not based on multiple sequence alignments; it does not use a substitution model and the distance measure is position specific. The NJ tree was generated using our own service ISTREE [25], which is freely available on http://istree.bioprotection.org/.

## Results

### Evolution of Egyptian H5N1 Strains by ISM-based Phylogeny

**Figure 1:**
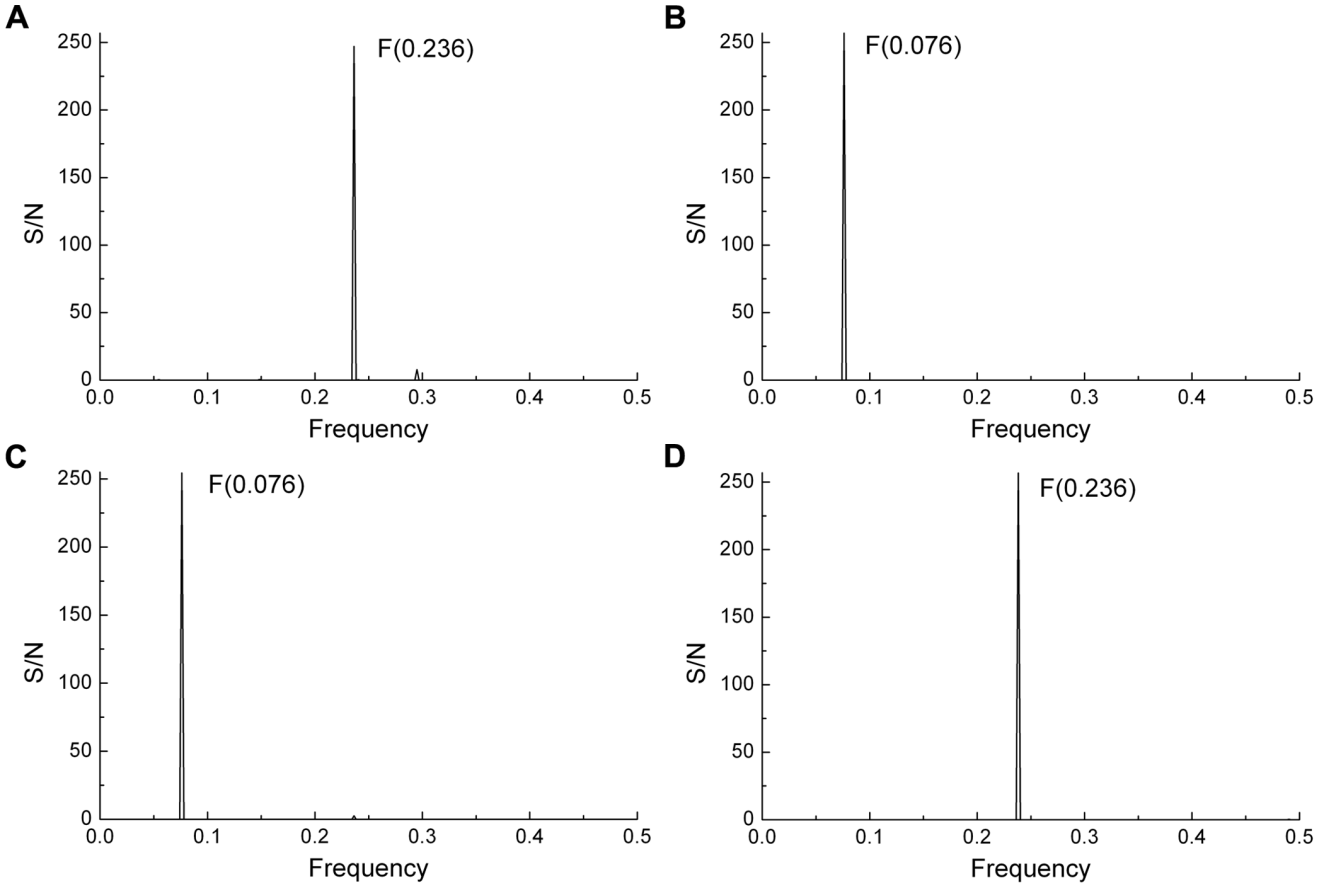
Comparison of informational spectra of seasonal H1N1 and Egyptian H5N1-HPAIV. (a) Cross-spectrum of HA1 of representative seasonal H1N1 viruses (n = 1576). (b) Cross-spectrum of all currently available Egyptian H5N1-HPAIV (n = 526). (c) Cross-spectrum of Egyptian H5N1-HPAIV isolated between 2006–2008 and (d) 2009–2011.

**Figure 2:**
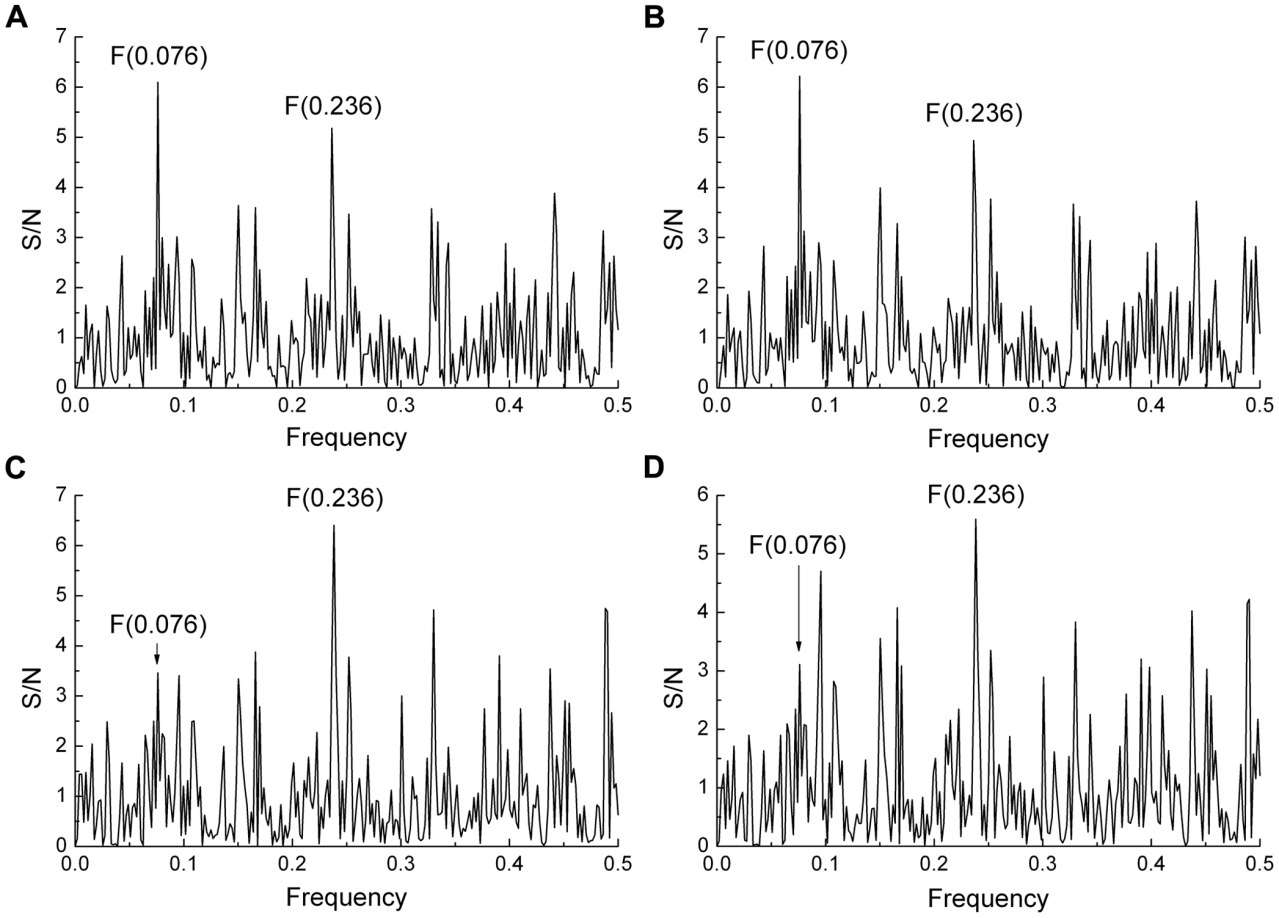
Comparison of informational spectra of H5N1-HPAIV from poultry and humans isolated 2006–2010. (a) A/chicken/Egypt/R1/2006, (b) A/Egypt/2763-NAMRU3/2006, (c) A/chicken/Egypt/1029/2010, (d) A/Egypt/N04434/2010.

The IS spectrum was calculated from 1576 publicly available HA1 sequences of seasonal H1N1 viruses from different years and geographic regions submitted to databases between May 2009 and November 2012. No sequences of pandemic H1N1 2009 were included in this set of viruses. The spectrum of a representative example (shown in Figure 1A) and the cross-spectrum of this large set of viruses showed a characteristic peak a the frequency F(0.236). This corresponds to our earlier findings with a smaller set of seasonal H1N1 viruses [5]. The cross-spectrum of all available H5N1-HPAIV HA1 sequences (N = 526) from Egypt showed a characteristic peak at F(0.076) (Figure 1B). The cross-spectra of the 2006–2008 Egyptian H5N1-HPAIV showed only the peak at F(0.076) (Figure 1C) whereas the 2009–2010 viruses showed a highly dominant peak at F(0.236) (Figure 1D). Thus Egyptian H5N1-HPAIV has also the characteristic peak of the seasonal H1N1 viruses. Figure 2 shows the individual IS of Egyptian H5N1-HPAIV obtained from poultry and humans in 2006 and 2010. The IS of the H5N1-HPAIV from 2006 (A/chicken/Egypt/R1/2006 (Figure 2A) and A/Egypt/2763-NAMRU3/2006 (Figure 2B)) have the dominant peak at the frequency F(0.076) typical for the H5N1-HPAIV using the avian receptor. The H5N1-HPAIV isolated in 2010 (A/chicken/Egypt/1029/2010 (Figure 2C) and A/Egypt/N04434/2010 (Figure 2D)) have the characteristic peak at the frequency F(0.236) typically found in the seasonal H1N1 interacting only with the human receptor. This suggests that H5N1-HPAIV circulating in Egypt in 2010 acquired increasingly properties of human influenza possibly with an increased propensity to react with the human receptor.

**Figure 3:**
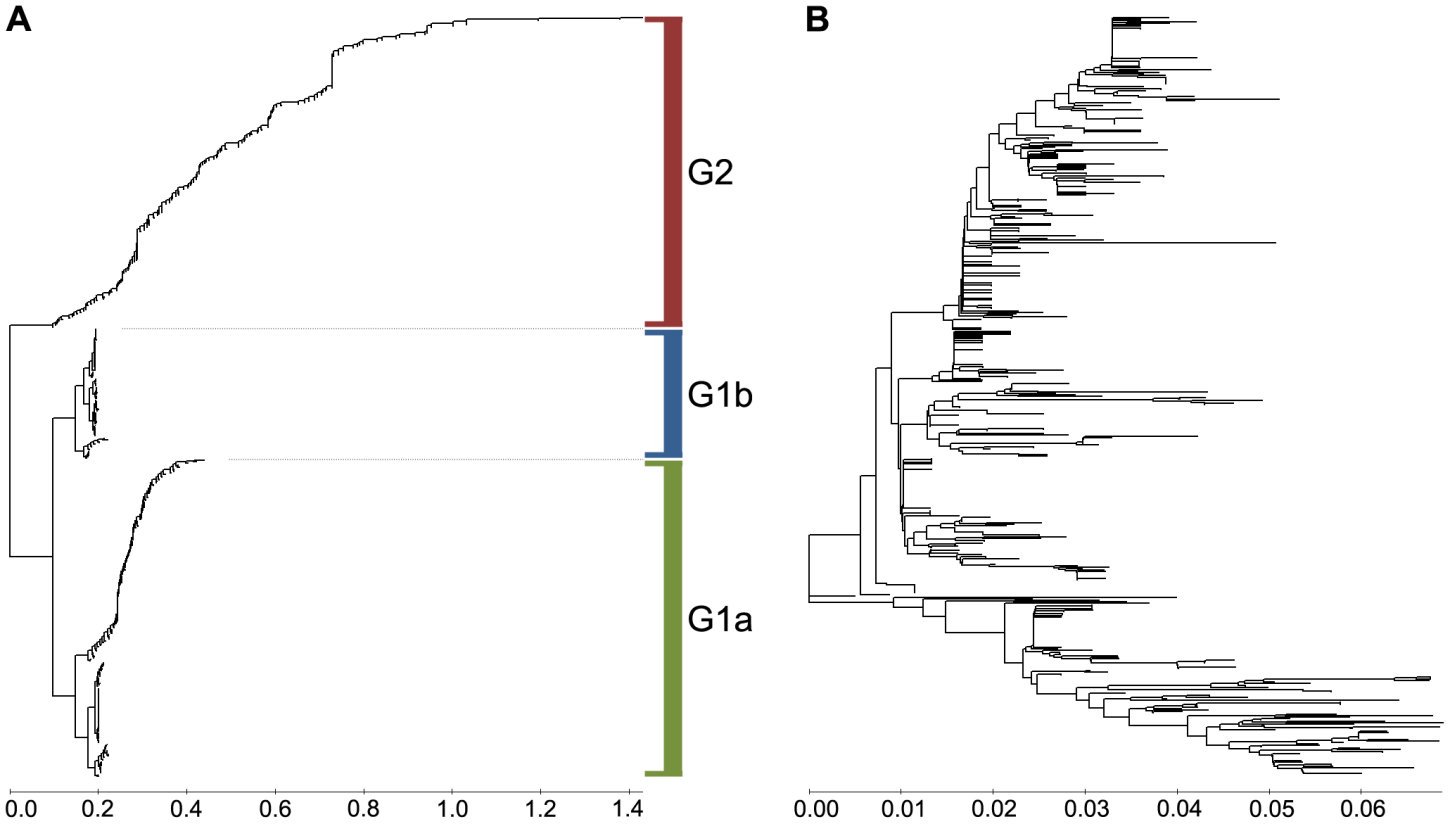
MSA-based and ISM-based phylogenetic analysis of H5N1-HPAIV isolated in Egypt. (a) The phylogenetic tree constructed using the IS-based method. (b) Phylogenetic tree constructed by the neighbor-joining method.

**Figure 4:**
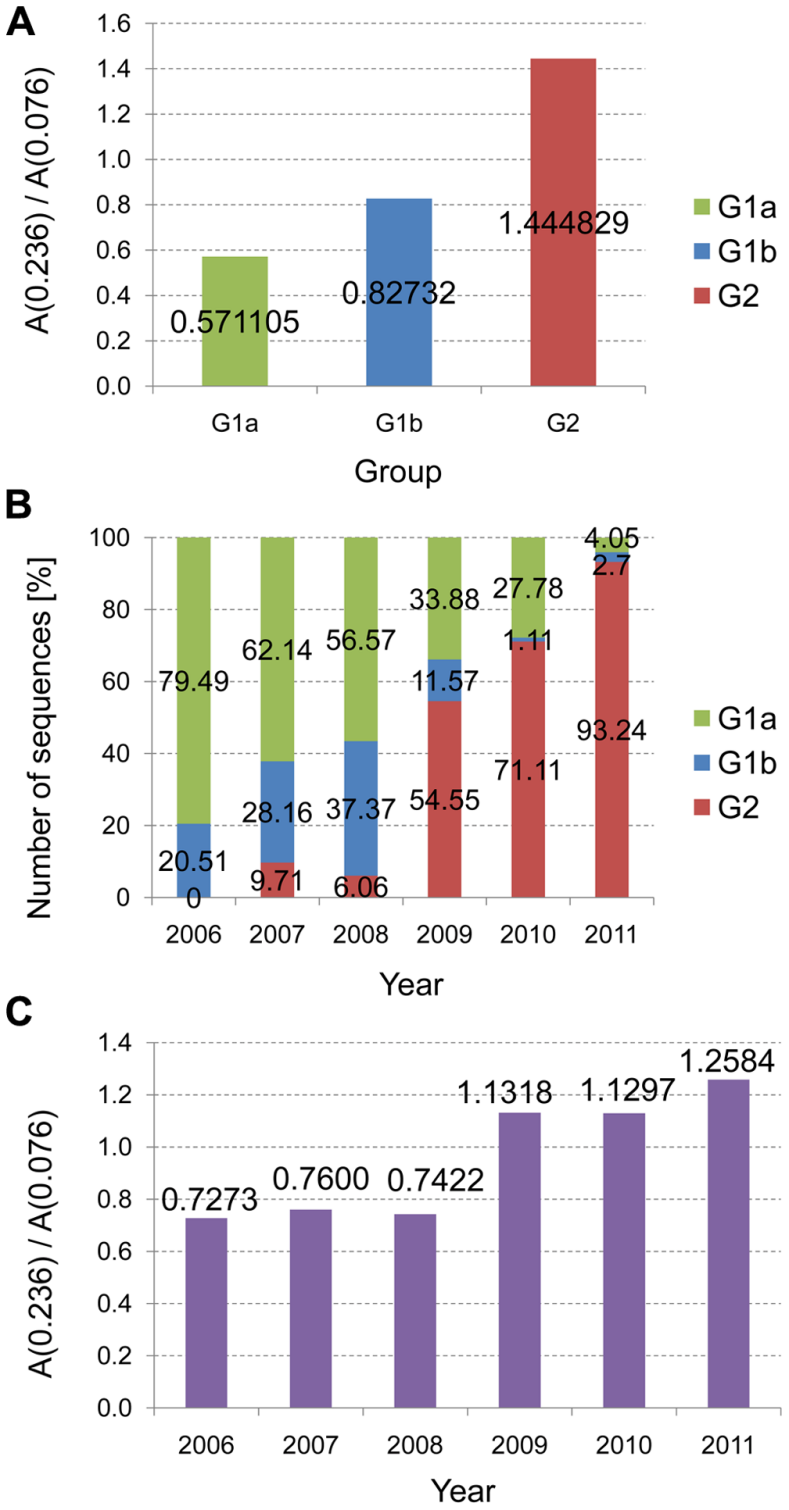
Distribution of A(0.236)/A(0.076) ratios by years for Egyptian H5N1-HPAIV isolated during 2006–2011. (a) The average values of A(0.236)/A(0.076) for G1 and G2 viruses. (b) Distribution of G1 and G2 viruses by years. (c) The average values of A(0.236)/A(0.076) by years.

**Figure 5:**
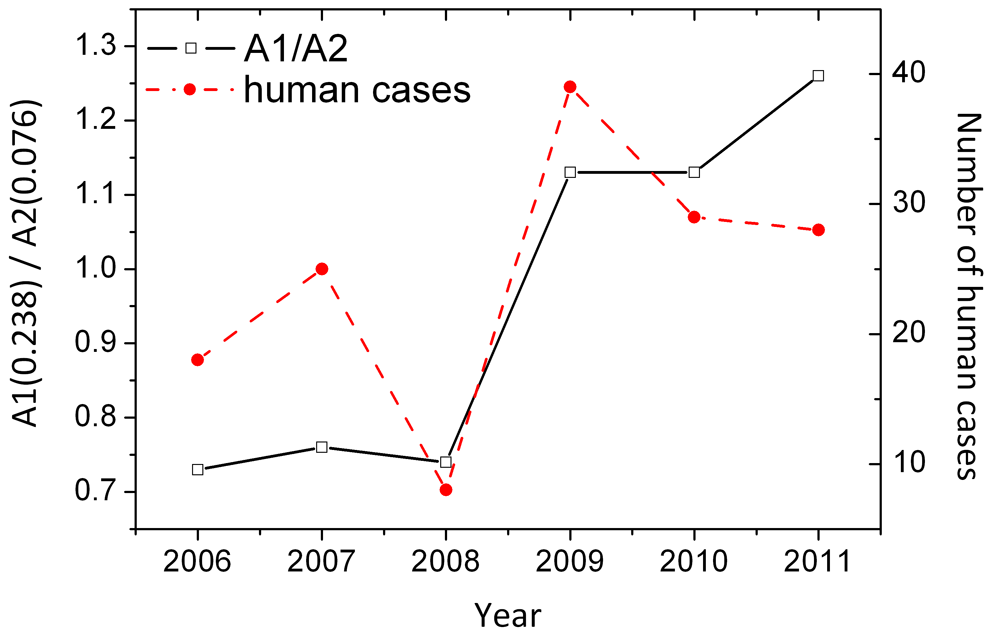
Correlation between A(0.236)/A(0.076) values of Egyptian H5N1-HPAIV and human cases. The total average A(0.236)/A(0.076) values and the number of human cases in Egypt between 2006 and 2010 are shown.

A phylogenetic tree was constructed using the A(0.236)/A(0.076) ratio as a distance matrix of the H5N1-HPAIV HA1 sequences as described in the Materials and Methods (Figure 3A and Figure S1 in high resolution). The tree structure revealed the emergence of two or three separate groups, G1a, G1b and G2. All G1 H5N1-HPAIV had an A(0.236)/A(0.076) <1 (average 0.646±0.224; Figure 4A) and all G2 viruses had an A(0.236)/A(0.076) >1 (average 1.445±0.311) (Figure 4A). The tree structure also showed a dramatic shift from 100% G1 viruses in 2006 to 93.2% G2 viruses in 2011, with a marked increase from 6% to 54% in 2008/2009 (Figure 4B). After their strongest turnout in 2008, the G1b viruses virtually disappeared in 2009/2010 (Figure 4B). Average values of the A(0.236)/A(0.076) ratio, calculated for all Egyptian H5N1-HPAIV for each year, showed a considerable increase of these values between 2006 and 2011 (Figure 4C). Furthermore 6.64% viruses with an A(0.236)/A(0.076) >1 were isolated between 2006 and 2008, and 69.82% of such viruses were isolated in the period 2009–2011. Thus, since 2006, more and more H5N1-HPAIV in Egypt acquired the characteristic IS feature (e.g. the receptor interaction pattern) of the seasonal H1N1. Figure 5 shows that there was a sudden increase in the number of human cases confirmed by World Health Organization (WHO) after 2008 [3]. Interestingly, in 2006–2008 there were 14 times more G1 viruses (n = 225) circulating than G2 viruses (n = 16), but in humans G1 viruses (n = 25) were found only 4.1 times more often than G2 viruses (n = 6). In 2009–2011 G2 viruses (n = 199) were only 2.3 times more prevalent than G1 viruses (n = 86), but they infected humans 9 times more often. Thus during both time periods G2 viruses infected humans significantly more often than would be expected from their overall prevalence (in humans and birds). Levels of significance were p<0.02 in 2006/2008 and further increased in 2009/2011 (p<0.007; 1-sided Pearson Chi-Square test of proportions). This is highly suggestive that viruses from the group G2 have an increased human tropism in comparison with viruses from the group G1. This further suggests that H5N1-HPAIV from group G2 have a higher affinity for humans and evolved towards receptor usage similar to that of seasonal H1N1 viruses.

Homology analysis of 526 Egyptian H5N1-HPAIV HA1 sequences revealed four group-specific amino acids (Table 2, Figure S4) that were consistently different between G1 and G2 viruses (the alignment of 526 Egyptian H5N1 HA1 sequences is given in Dataset S2). All of G1 viruses had the amino acids D43, S120, (S,L)129 and I151 whereas >94% of G2 viruses had N43, (D,N)120, 129Δ and T151 (amino acid positions according to H5 numbering; Table S2). While Table S2 lists another 18 positions in which G1 and G2 viruses differ, positions 43, 120, 129 and 151 were unique in their power to discriminate between G2 and G1 sequences. Both in poultry viruses and in human viruses the four G2-typical amino acids steadily increased with time and were mostly acquired after 2008 (Table 3).

**Table 2:** Residues that are specific for H5N1-HPAIV HA1 groups G1 and G2 (Figure 1A) and mutations that significantly increase the A(0.236)/A(0.076) ratio.

	Group G1	Group G2
**Group-specific residues**	D43, S120, (S,L)129, I151	N43, (D,N)120, 129del, T151
**Mutations that significantly increase A(0.236)/A(0.076) ratio**	P74S, H110R, A127T, F143Y, K153D, S188K, S223(N,I), S234P, G272S, N275S	

Residue positions correspond the H5N1 numbering.

**Table 3:** Number of G2 specific amino acid substitutions which are acquired before and after 2009.

Mutation	Number of mutation (2006–2008)	Number of mutation (2009–2011)
D43N	15 (7%)	200 (93%)
S120(N,D)	23 (12%)	196 (88%)
S129Δ	13 (6%)	191 (94%)
T151I	13 (6%)	207 (94%)

**Figure 6:**
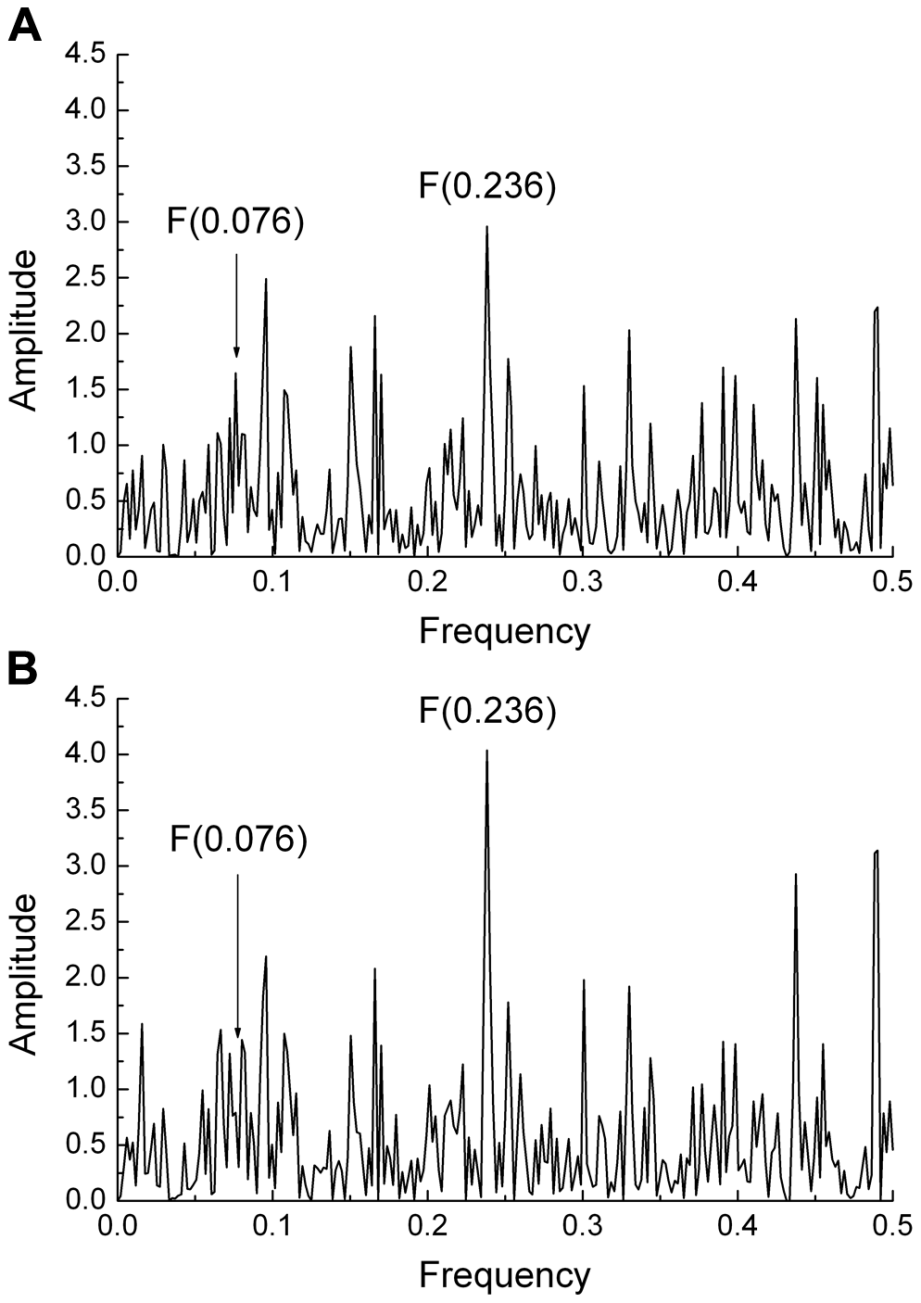
Effect of mutations on informational spectra of the Egyptian H5N1-HPAIV A/Egypt/N04434/2010. Effect of mutations P74S, H110R, A127T, F143Y, K153D, S188K, S223(N,I), S234P, G272S, N275S from Table 2) on IS of HA1 from human H5N1 virus A/Egypt/N04434/2010. (a) IS of the unmutated HA1 protein, (b) IS of HA1 with mutations.

The IS based phylogenetic tree shows that within the G2 group even after acquisition of the changed amino acid pattern, the A(0.236)/A(0.076) ratio continued to increase from 1.13 in 2009 to an average value of 1,26 in 2011. The continued increase in this ratio was the result of a number of additional mutations that accumulated with time. Table 2 shows a selection of mutations which increased the value of the A(0.236)/A(0.076) ratio and that were found in at least 2 strains of Egyptian viruses analyzed. Interestingly, most of these mutations were also found in G1 viruses, but G1 viruses have one, two or a maximum three of these mutations from Table 2, which was not enough for the increase of A(0.236)/A(0.076) above 1. In order to evaluate the cumulative effect of these mutations, we compared the IS of the most recent Egyptian H5N1-HPAIV HA1 sequence publicly available (A/Egypt/N04434/2010; collected in March 2010) (Figure 6A) with the IS of this HA1 with all mutations from the second line in Table 2 (Figure 6B). The cumulative effect of these mutations dramatically increased the A(0.236)/A(0.076) ratio from 1.79 for the native protein (Figure 6A) to 5.11 for the mutated protein (Figure 6B). This suggests that the acquisition of additional mutations (that are already found in G1 viruses) by G2 viruses bears further potential for human adaptation, whereas the G1 viruses do not have this potential. Therefore, the amino acid substitutions given in Table 2 may represent valuable molecular markers to further monitor the changing epidemiology of H5N1-HPAIV infections towards human adaptation in Egypt.

### Comparison of the ISM-based and the MSA-based Phylogeny

**Figure 7:**
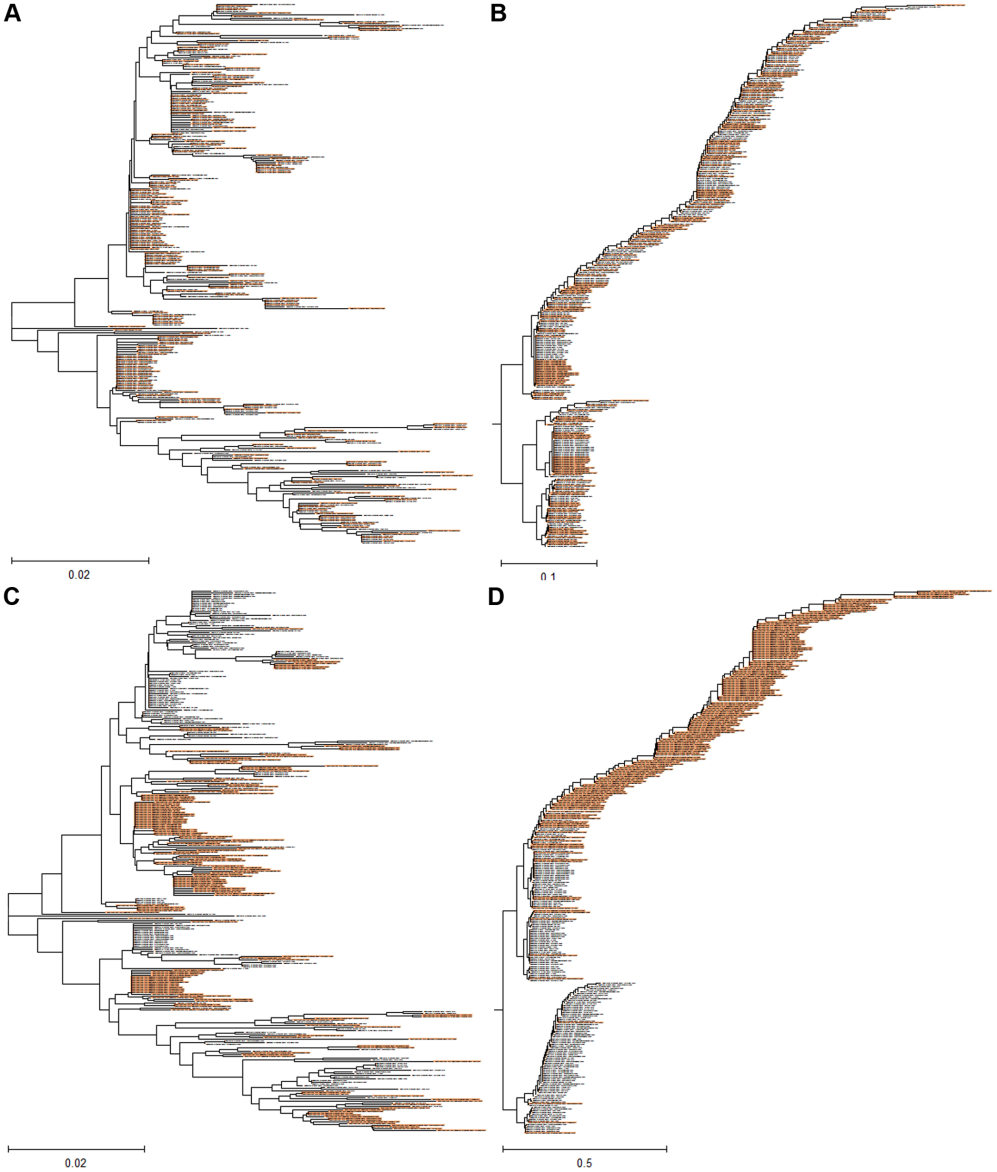
Comparison of sensitivity of MSA-based and ISM-based phylogenetic tree to mutations. Mutations which are potentially important for human tropism of Egyptian H5N1-HPAIV are included to test the sensitivity of MSA- and ISM-based approach to mutations. (a) The MSA tree and (b) the ISM tree for all 311 unmutated H5N1-HPAIV HA1 sequences belonging to G1 viruses; (c) the MSA tree of panel (a) in which each second sequence was mutated and (d) the ISM tree which corresponds to the mutated and unmutated sequences presented in panel (c). H5N1-HPAIV HA1 sequences selected for mutations D43N, S120D, S129Δ and I151T are in red.

Figure S2 and Figure 3B show the phylogenetic tree for Egyptian H5N1-HPAIV viruses, based on the NJ and the maximum likelihood method. Both trees have a very similar structure confirmed by high bootstrap values. Similar to the IS-based tree (Figures 3A and Figure S1), most of the G2 viruses emerge from a single common branch in these conventional trees. Therefore, we compared the sensitivity of the multiple sequences alignment-based (MSA) and IS-based phylogenetic algorithms to detect the above functionally important mutations. Figure 7A and B present the MSA and IS-based trees of the HA1 sequences of all 311 G1 viruses. We introduced the four amino acids (43N, 120D, 129del, 151T) characteristic of G2 viruses into every second sequence of the selected set. Figure 7C and D shows that in contrast to the MSA-based tree which is not sensitive to the mutations, the IS-based tree clearly segregated the mutated sequences into the G2 cluster.

Furthermore, we identified 32 non-conserved positions in the Egyptian sequence dataset (dataset S1, S2) by comparison with the oldest Egyptian H5N1-HPAIV isolate in the dataset. These positions were substituted by selected amino acids (I = 0.00000 Ry, P = 0.01979 Ry, K = 0.03718 Ry, Y = 0.05159 Ry, Q = 0.07606 Ry, T = 0.09408 Ry, D = 0.12630 Ry) with EIIP values, which are more or less evenly distributed across the EIIP space (see Table 1). Each of the 32 non-conserved amino acids was replaced by each of the above selected amino acids. The impact of these monosubstitutions on the A(0.236)/A(0.076) ratio was calculated for each position. Figure S3 shows the variation of the A(0.236)/A(0.076) ratio as a function of the substitutions in the 32 positions. Table S1 shows the average value of the change in A(0.236)/A(0.076) ratio for each of the above amino acids when introduced into each of the non-conserved positions. Both of these supplementary data show that the positions considerably differ in their sensitivity to substitutions by the different amino acids (Figure S3); and that the impact of each amino acid greatly differs depending on the position (Table S1).

We also generated the phylogenetic tree for each set of 32 sequences obtained by the monosubstitution by each of the selected amino acids (I, P, K, Y, Q, T, D) in the 32 non-conserved positions of HA. The results presented in Figures S5A – S5G show that the tree structure of the IS-tree is strongly influenced by the monosubstitutions. In contrast, Figures S5H – S5N show that the MSA-based trees for the same substituted sequences is not sensitive to the single amino acid substitutions.

## Discussion

Here we developed an IS-based phylogenetic algorithm based on the ratio of the amplitude at selected important frequencies as a distance measure. The algorithm can be used to follow the evolution of the interactive profile of viruses. We have used the algorithm to analyze H5N1-HPAIV circulating in Egypt from 2006 to 2011 using the A(0.236)/A(0.076) ratio as a distance measure. This analysis revealed that all HA1 sequences from Egyptian H5N1-HPAIV which are available in the public databases cluster in two groups G1 and G2 (Figure 3A and Figure S1). Although the important G2 group was identified as a separate cluster by both the IS-based and the conventional phylogenetic tree, the conventional tree did not reveal particular mutations that are associated with the evolution towards typical features of seasonal (human) influenza. Our IS-based phylogenetic approach is generic as it can utilize in the distance matrix any ratio of amplitudes of two frequencies that are of interest with respect to the interaction profile of a (viral) protein.

One of these groups (G2) shows a very dramatic propensity towards high A(0.236)/A(0.076) ratios whereas only one subgroup of G1 showed a weak propensity while the other did not. We showed that the A(0.236)/A(0.076) ratio increases with time as a result of more viruses having higher A(0.236)/A(0.076) ratios. The high A(0.236)/A(0.076) ratios result mainly from four amino acid mutations that are found in the G2 viruses but not in G1 viruses (N84, 120(N,D), 129Δ and T151I). Among all amino acid differences between G1 and G2 sequences these are unique in their power to discriminate between G1 (>92%) and G2 (>94%) viruses.

Recently Watanabe and coworkers reported that H5N1-HPAIV with three of these mutations (S120N, 129Δ and I151T) or even with the latter two alone, have an increased affinity for α2,6-linked sialic acid (SA) [26] and an increased potential for human H5N1-HPAIV infection. These authors further showed that the 129S insertion and reverse mutation T151I decreased α2,6 SA binding. In contrast, each of these amino acid substitutions alone had no favorable effect on binding to the human α2,6 SA. All viruses reported by Watanabe and coworkers as BI and BII sublineages, characterized by the above mutations (S120N, 129Δ and I151T) [26], belong to G2 viruses with the preferred interacting profile similar to that of seasonal H1N1 viruses.

Since the peaks at F(0.236) and at F(0.076) are typical for human seasonal H1N1 and for H5N1-HPAIV respectively [5], we interpreted the increasing A(0.236)/A(0.076) ratio as a progressive evolution of H5N1-HPAIV towards human tropism with a higher selectivity for the human receptor and probably an increased risk of human infections [5].

This is indeed supported by our observation that viruses with high A(0.236/A(0.076) ratios were more likely to infect humans. Already in 2006–2008 G2 viruses were significantly (p<0.02) more often found in humans than was expected from their overall prevalence. In 2009–2011 this became highly significant (p<0.007) with 9 times more G2 viruses in humans compared to only 2.3 fold relative overall prevalence. This is the more interesting since there are no or only low human to human transmissions and most viruses in humans dead-end. As a result viruses with the double human/poultry tropism so far did not have the opportunity to be selected for their human tropism in humans.

Several other lines of observations have subsequently confirmed this:

- Kayali and coworkers identified a new sublineage of H5N1-HPAIV within clade 2.2.1 named “Egypt-G” [27]. These viruses appeared in Egypt in 2009, and seemed to be more adapted to human-to-human transmission. Their analysis also revealed that these new viruses were less virulent and may even cause asymptomatic infections and that the silent spread of H5N1-HPAIV in humans providing ample opportunities to further accelerate viral evolution. According to these authors, most Egyptian H5N1-HPAIV belonged to the sublineages Egypt E-F and circulated exclusively in poultry. In contrast to Egypt-G viruses, there was no evidence of human infections with the latter viruses. All viruses of the sublineages Egypt-G belonged to our group G2 and all Egypt E-F strains belonged to G1 (Figure 3A).
- The typical G2 mutations are rare in H5N1-HPAI viruses. Analysis of all H5N1-HPAIV from Asia, Europe, America and Africa from NCBI and GISAID databases, revealed only 12 H5N1-HPAIV with the mutation 129Δ, and only 9 viruses with the combined mutations 129Δ and I151T. The 129Δ deletion did not occur in H5N1-HPAIV originally introduced into Egypt in 2006 [28], [29]. Isolates from non-human mammals likewise did not reveal the 129Δ. Interestingly, 129Δ viruses were found in the majority of investigated human infections in Egypt in 2009 [30] and this deletion has been found in all human seasonal H1N1 and H3N2 viruses [30].
- Similarly, I141T is also seen in all human seasonal H1N1 and H3N2 viruses [30], while S120(N,D) is unique to Egyptian H5N1-HPAIV.
- The mutations recently reported by Kawaoka et al. [31] (N224K, Q226L, T318I except N158D) which facilitated transmission by droplets in ferrets increased the A(0.236)/A(0.076) ratio when introduced for instance into the Egyptian G1 virus selected by WHO as a new vaccine candidate (A/Egypt/N03072/2010). N158 is present in 68% of all Egyptian H5N1-HPAIV both in G1 and G2 viruses, including very early strains isolated in 2006. This mutation has no effect on the A(0.236)/A(0.076) ratio, but its high prevalence further underlines the high pandemic potential of Egyptian H5N1-HPAIV.
- There are numerous other studies which investigated effects of mutations in H5N1-HPAIV HA1 on its binding to α2,3 SA and α2,6 SA **(**reviewed in [32]). In Table 4 are given mutations which increase binding of H5N1-HPAIV HA1 to human receptor [28], [29], [33]–[38], but none of these mutations were found in G1 nor in G2 viruses which supposedly have an increased human tropism. The only exception is the substitution S223N which facilitates α2,6 SA binding. This mutation also increases the A(0.236)/A(0.076) ratio but is present in <1% of Egyptian H5N1-HPAIV (Table 2).

**Table 4:** Mutations that increase binding of H5N1-HPAIV HA1 to the human α2,6-linked sialic acid.

Mutations	Reference
S223N, D183G, M E186G, Q192R, Q222L, G224S	30
N182K, Q192R	31
N182K, Q22L, G224S	32
N183G	33
D94N	34
(A,I,P,S,T)86V, (N,D)108S, (Q,H,I)122(L,N), A156(T,S), K112(E,R),A163T	35
A86V, A156T	36
H125Q, V148A, N154D, Y157H, T159A, Y164C, W176R	37

All of the above observations and experimental results provide strong support that our phylogenetic algorithm based on a distance matrix of the A(0.236)/A(0.076) ratio uniquely captures an ongoing evolution of Egyptian H5N1-HPAIV towards human tropism and probably a higher infectivity for humans. This evolution is characterized by highly specific H5N1-HPAIV HA1 mutations, which we identified with a totally independent and unrelated (computational) method based on a parameter that we described already before the above publications, in particular before the report of Watanabe which experimentally confirms the human tropism of three of the four mutations that are most typical for G2 viruses. In our 2009 publication Egyptian sequences only from 2006 and 2007 were available including only 35 G2 sequences.

Recently, five H5N1-HPAIV HA1 sequences from Indonesia 2007 became available all of which exhibited the two mutations 129del and I151T and 3 strains have in addition N43 (GISAID), characteristic for the G2 Egyptian viruses but none had the forth substitution or any other Egyptian mutation that increases affinity to the human receptor. Interestingly, Indonesian viruses do not share the frequency F(0.236) with seasonal H1N1 or Egyptian G2 viruses. In the Indonesian H5N1-HPAIV the typical G2 mutations do not increase the A(0.236)/A(0.076) ratio. This suggests that in contrast to Egyptian H5N1-HPAI viruses the published Indonesian viruses did not evolve towards a human interacting profile but no recent sequences are available.

Recently two neutralizing human antibodies were mapped to aa125–129 [39]–[41], within the domain 99–133 which we identified as essential for recognition and targeting of Egyptian H5N1-HPAIV to human receptor [5] and which is highly conserved in all H5N1-HPAIV clades with the exception of Egyptian G2 viruses. In this H5N1-HPAIV HA1 region are located two of four amino acid substitutions (N/D120 and S120Δ) which are essential for increased human tropism of Egyptian H5N1-HPAIV (Table 2). This strongly suggests that reported human anti-H5N1-HPAIV antibodies will not be effective against G2 viruses.

More importantly, however, in 2011 the WHO selected A/Egypt/N03072/2010 as a new candidate vaccine virus [42]. This virus does not contain N/D120 possibly compromising its effectiveness against G2 viruses. As no new Egyptian viruses have been made available on public data bases since 2010 the importance of the G2 viruses may have been overlooked.

Of note is that G2 viruses are no longer confined to Egypt: 3 of 5 H5N1-HPAIV isolated in Israel in 2010 and 2011 have mutations corresponding to Egyptian G2 viruses. Prior to 2010 all H5N1-HPAIV in Israel were G1 viruses.

The mechanism underlying the emergence of H5N1-HPAIV in Egypt with both α2,6 SA and α2,3 SA binding affinity is not clear. One possible explanation is that this accelerated evolution of H5N1 [43], [44] is driven by the antigenic drift which is initiated by the nationwide poultry vaccination campaign in Egypt with poorly matching vaccines [45]. In conclusion, we analyzed here the extensive evolution of HA1 from H5N1-HPAIV in Egypt towards the human interacting profile using a novel phylogenetic approach based on distance measures derived from the IS method. The spread by asymptomatic infections in humans [2] as well as in wild birds [46] of H5N1-HPAI viruses in Egypt that seem to increasingly adapt to humans represents a major pandemic threat. This underlines an urgent need to make sequences of H5N1-HPAIV available for monitoring the evolution of molecular determinants of virulence of H5N1-HPAI viruses.

## Supporting Information

Figure S1 **High resolution of the phylogenetic tree of Egyptian H5N1-HPAIV based on ISM in** **Figure 3a** **.**
(TIF)

Figure S2 **High resolution of the phylogenetic tree of Egyptian H5N1-HPAIV constructed by the maximum-likelihood method.**
(TIF)

Figure S3 **Sensitivity of A(0.236)/A(0.076) ratio to mutations in non-conserved positions of H5N1-HPAIV HA1.** A(0.236)/A(0.076) ratio as a function of single substitutions with I, P, K, Y, Q, T, D in each of the 32 non-conserved positions of H5N1 HA1 (GenBank: strain designation ABW37431).
(TIF)

Figure S4 **Identification of non-conserved positions over the whole sequence of HA1 from G1 and G2 viruses.** Red/black/green arrow for high/medium/small percentage difference.
(TIF)

Figure S5 **Influence of position and type of mutation HA1 from H5N1-HPAIV on MSA-based and ISM-based phylogenetic tree.** Phylogenetic tree for each set of 32 sequences obtained by the introduction of each selected amino acid (I, P, K, Y, Q, T, D) in 32 non-conserved positions of HA. (A) - (G), phylogenetic trees based on ISM, (H) – (N) phylogenetic trees constructed by the neighbor-joining method. The wild-type sequence (ABW37431/A/chicken/Egypt/R1/2006) is highlighted in each tree. All other sequences have a single substitution by one of the above amino-acids (panel A,H: amino acid substitution by I; panel B,I: substitution by P etc. …) in the different non-conserved positions. The substitution is given as part of the strain name.
(TIF)

Table S1 **Average and standard deviation of the A(0.236)/A(0.076) values.** A(0.236)/A(0.076) ratios correspond to single amino-acid substitution in each of the 32 non-conserved positions by I, P, K, Y, Q, T, D. Each value corresponds to a tree in Figure S5, panel A-G.
(DOC)

Table S2 **Distribution of group-specific amino-acids between G1 and G2 viruses.**
(DOC)

Dataset S1 **FASTA-formatted file of the 526 sequences of Egyptian H5N1-HPAIV.** Viruses from 2006 to 2011 published on NCBI and GISAID are included.
(TXT)

Dataset S2 **Alignment of HA1 sequences of the 526 Egyptian H5N1-HPAIV included in this study.**
(TXT)
