# Supplementary figures and images for: Novel Phylogenetic Algorithm to Monitor Human Tropism in Egyptian H5N1-HPAIV Reveals Evolution toward Efficient Human-to-Human Transmission
Source: PLoS One. 2013 Apr 26;8(4):e61572. doi: 10.1371/journal.pone.0061572 (PMC3637272; doi:10.1371/journal.pone.0061572)

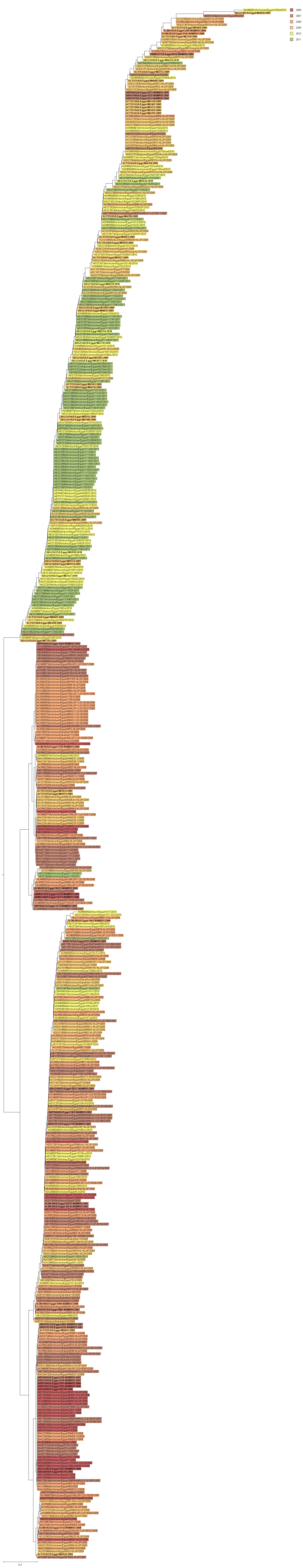

Supplement: Figure S1 — High resolution of the phylogenetic tree of Egyptian H5N1-HPAIV based on ISM in Figure 3a . (TIF) [file pone.0061572.s001.tif]

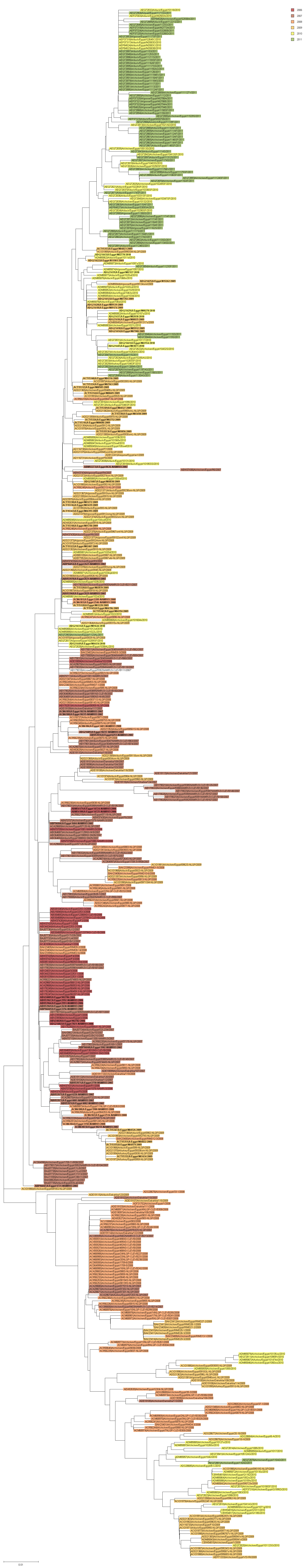

Supplement: Figure S2 — High resolution of the phylogenetic tree of Egyptian H5N1-HPAIV constructed by the maximum-likelihood method. (TIF) [file pone.0061572.s002.tif]

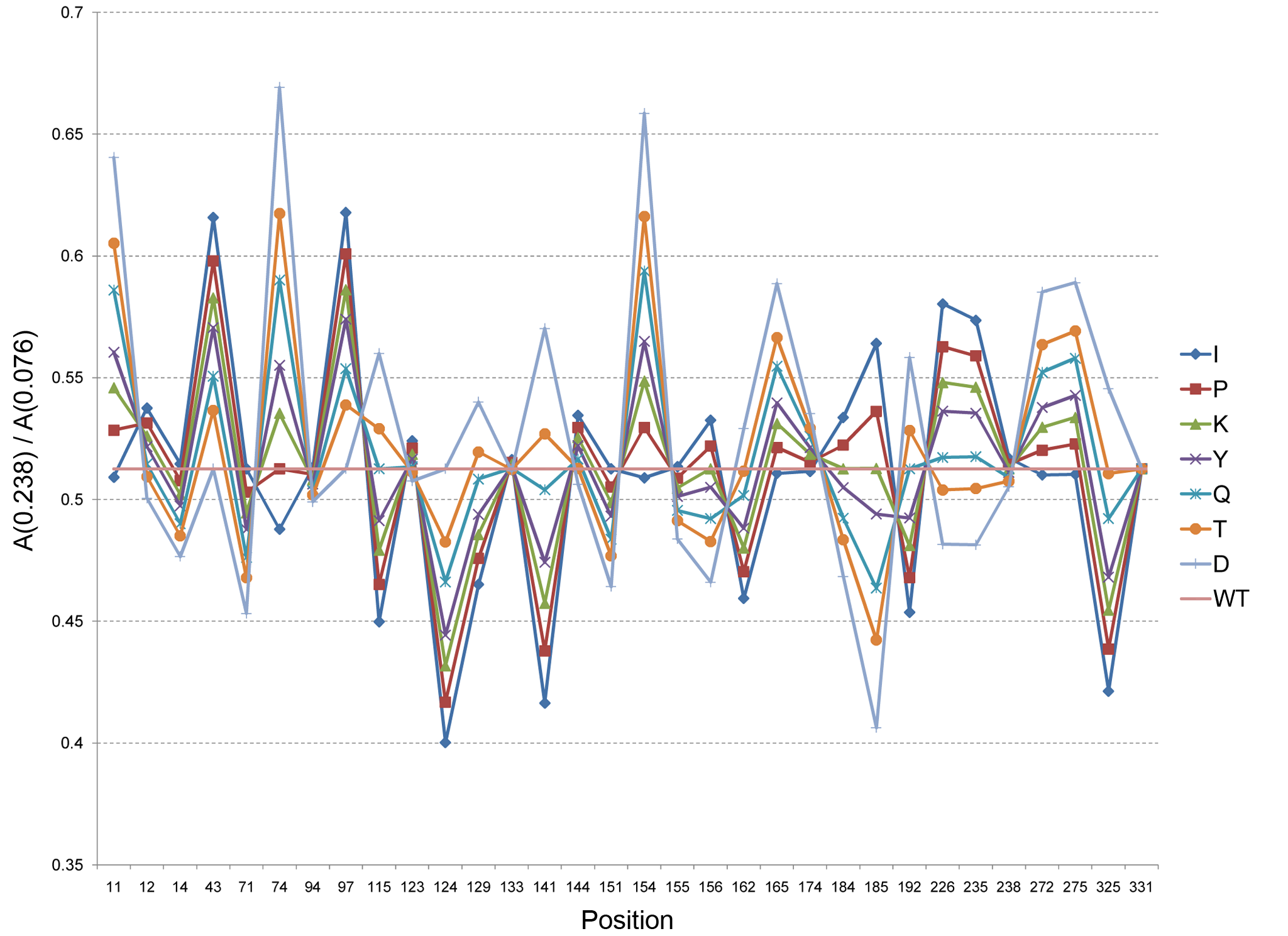

Supplement: Figure S3 — Sensitivity of A(0.236)/A(0.076) ratio to mutations in non-conserved positions of H5N1-HPAIV HA1. A(0.236)/A(0.076) ratio as a function of single substitutions with I, P, K, Y, Q, T, D in each of the 32 non-conserved positions of H5N1 HA1 (GenBank: strain designation ABW37431). (TIF) [file pone.0061572.s003.tif]

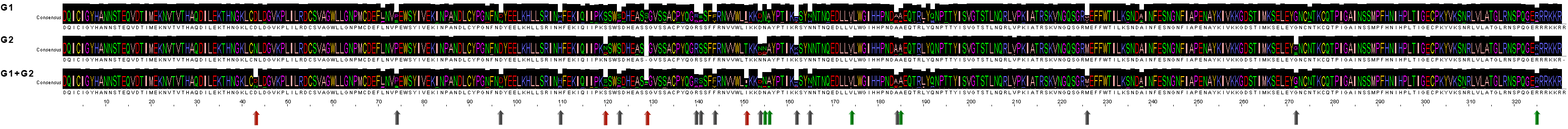

Supplement: Figure S4 — Identification of non-conserved positions over the whole sequence of HA1 from G1 and G2 viruses. Red/black/green arrow for high/medium/small percentage difference. (TIF) [file pone.0061572.s004.tif]

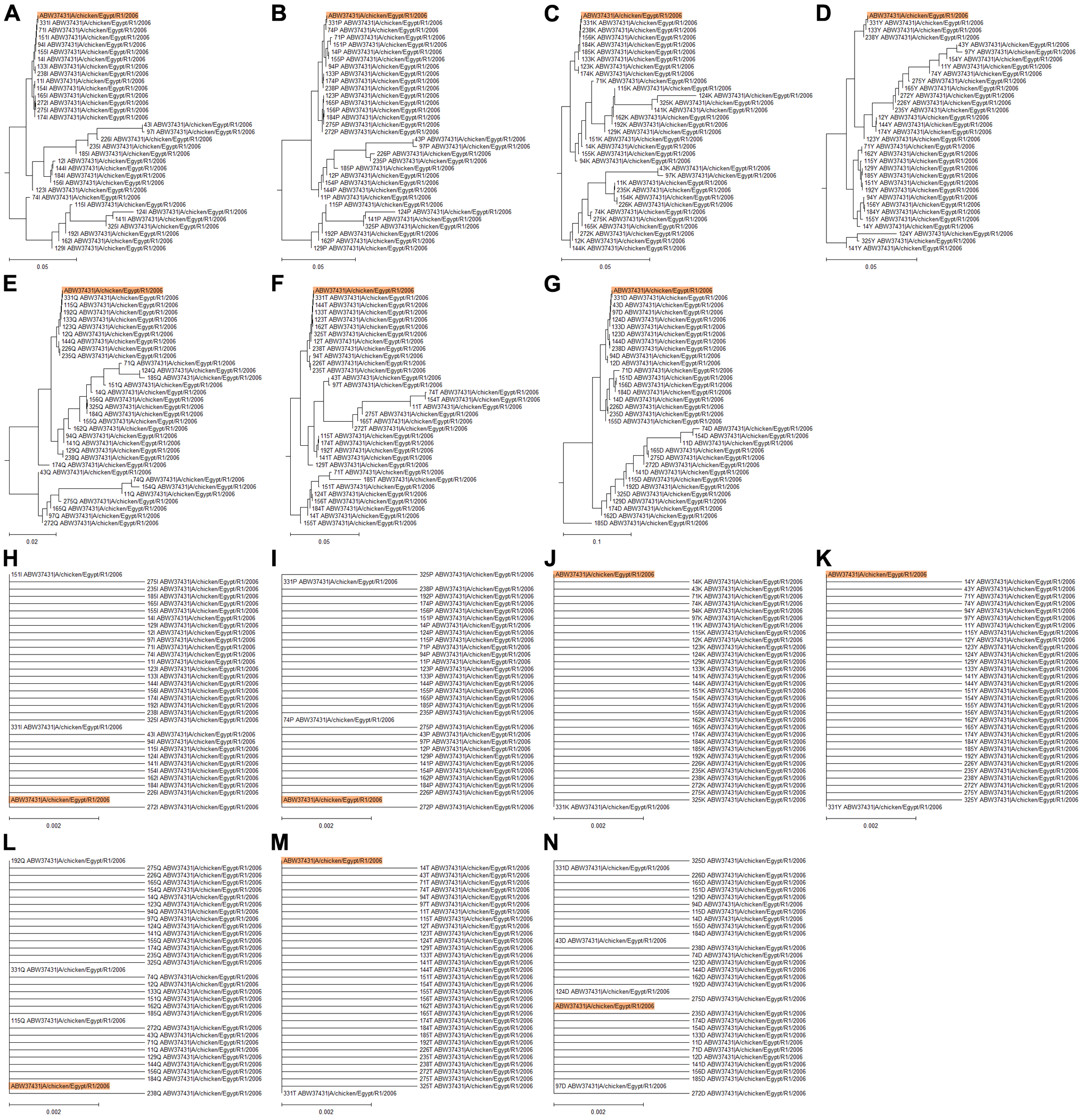

Supplement: Figure S5 — Influence of position and type of mutation HA1 from H5N1-HPAIV on MSA-based and ISM-based phylogenetic tree. Phylogenetic tree for each set of 32 sequences obtained by the introduction of each selected amino acid (I, P, K, Y, Q, T, D) in 32 non-conserved positions of HA. (A) - (G), phylogenetic trees based on ISM, (H) – (N) phylogenetic trees constructed by the neighbor-joining method. The wild-type sequence (ABW37431/A/chicken/Egypt/R1/2006) is highlighted in each tree. All other sequences have a single substitution by one of the above amino-acids (panel A,H: amino acid substitution by I; panel B,I: substitution by P etc. …) in the different non-conserved positions. The substitution is given as part of the strain name. (TIF) [file pone.0061572.s005.tif]
