# Supplementary material for: Novel Phylogenetic Algorithm to Monitor Human Tropism in Egyptian H5N1-HPAIV Reveals Evolution toward Efficient Human-to-Human Transmission
Source: PLoS One. 2013 Apr 26;8(4):e61572. doi: 10.1371/journal.pone.0061572 (PMC3637272; doi:10.1371/journal.pone.0061572)
Supplement: Table S1 — Average and standard deviation of the A(0.236)/A(0.076) values. A(0.236)/A(0.076) ratios correspond to single amino-acid substitution in each of the 32 non-conserved positions by I, P, K, Y, Q, T, D. Each value corresponds to a tree in Figure S5, panel A-G. (DOC) [file pone.0061572.s006.doc]

**Table S1.** **Average and standard deviation of the A(0.236)/A(0.076) values.**

A(0.236)/A(0.076) ratios correspond to single amino-acid substitution in each of the 32 non-conserved positions by I, P, K, Y, Q, T, D. Each value corresponds to a tree in Figure S5, panel A-G.

| **Amino acid** | **Average** | **Standard Deviation** |
| --- | --- | --- |
| I | 0.510654458 | 0.050169727 |
| P | 0.511998690 | 0.040066798 |
| K | 0.513499517 | 0.033442625 |
| Y | 0.514971871 | 0.030692419 |
| Q | 0.517952601 | 0.033572762 |
| T | 0.520539452 | 0.040824044 |
| D | 0.526009115 | 0.059402374 |
