# Supplementary material for: Novel Phylogenetic Algorithm to Monitor Human Tropism in Egyptian H5N1-HPAIV Reveals Evolution toward Efficient Human-to-Human Transmission
Source: PLoS One. 2013 Apr 26;8(4):e61572. doi: 10.1371/journal.pone.0061572 (PMC3637272; doi:10.1371/journal.pone.0061572)
Supplement: Table S2 — Distribution of group-specific amino-acids between G1 and G2 viruses. (DOC) [file pone.0061572.s007.doc]

**Table S2. Distribution of group-specific amino-acids between G1 and G2 viruses.**

|  | **Group G1** | **Group G2** |
| --- | --- | --- |
| **Group-specific residues with high percentage difference > 90%** | D43 (99%) | N43 (98%) |
|  | S120 (94%) | (D,N)120 (34%,60%) |
|  | (S,L)129 (71%,21%) | 129del (99%) |
|  | I151 (92%) | T151 (99%) |
| **Group-specific residues with medium percentage difference 35% - 55%** | S74 (40%) | P74 (100%) |
|  | N97 (43%) | D97 (100%) |
|  | R110 (39%) | H110 (100%) |
|  | P123 (38%) | S123 (99%) |
|  | G140 (35%) | R140 (98%) |
|  | P141 (42%) | S141 (98%) |
|  | Y144 (40%) | F144 (99%) |
|  | H165 (39%) | N165 (100%) |
|  | E184 (41%) | A184 (92%) |
|  | (V,I)226 (39%,17%) | M226 (99%) |
|  | G272 (99%) | S272 (40%) |
| **Group-specific residues with small percentage difference < 30%** | D154 (65%) | N154 (61%) |
|  | N155 (98%) | D155 (28%) |
|  | T156 (25%) | A156 (98%) |
|  | (I,E)162 (14%,10%) | (R,K)162 (43%,56%) |
|  | V174 (97%) | I174 (5%) |
|  | A185 (79%) | T185 (6%) |
|  | R325 (81%) | K325 (26%) |
